# Development of a machine learning model to predict mild cognitive impairment using natural language processing in the absence of screening

**DOI:** 10.1186/s12911-022-01864-z

**Published:** 2022-05-12

**Authors:** Robert B. Penfold, David S. Carrell, David J. Cronkite, Chester Pabiniak, Tammy Dodd, Ashley MH Glass, Eric Johnson, Ella Thompson, H. Michael Arrighi, Paul E. Stang

**Affiliations:** 1 Kaiser Permanente Washington Health Research Institute, 1730 Minor Ave., Suite 1600, Seattle, WA 98101 USA; 2 Janssen Research and Development, LLC, Raritan, USA

**Keywords:** NLP, MCI, Dementia, Early identification

## Abstract

**Background:**

Patients and their loved ones often report symptoms or complaints of cognitive decline that clinicians note in free clinical text, but no structured screening or diagnostic data are recorded. These symptoms/complaints may be signals that predict who will go on to be diagnosed with mild cognitive impairment (MCI) and ultimately develop Alzheimer’s Disease or related dementias. Our objective was to develop a natural language processing system and prediction model for identification of MCI from clinical text in the absence of screening or other structured diagnostic information.

**Methods:**

There were two populations of patients: 1794 participants in the Adult Changes in Thought (ACT) study and 2391 patients in the general population of Kaiser Permanente Washington. All individuals had standardized cognitive assessment scores. We excluded patients with a diagnosis of Alzheimer’s Disease, Dementia or use of donepezil. We manually annotated 10,391 clinic notes to train the NLP model. Standard Python code was used to extract phrases from notes and map each phrase to a cognitive functioning concept. Concepts derived from the NLP system were used to predict future MCI. The prediction model was trained on the ACT cohort and 60% of the general population cohort with 40% withheld for validation. We used a least absolute shrinkage and selection operator logistic regression approach (LASSO) to fit a prediction model with MCI as the prediction target. Using the predicted case status from the LASSO model and known MCI from standardized scores, we constructed receiver operating curves to measure model performance.

**Results:**

Chart abstraction identified 42 MCI concepts. Prediction model performance in the validation data set was modest with an area under the curve of 0.67. Setting the cutoff for correct classification at 0.60, the classifier yielded sensitivity of 1.7%, specificity of 99.7%, PPV of 70% and NPV of 70.5% in the validation cohort.

**Discussion and conclusion:**

Although the sensitivity of the machine learning model was poor, negative predictive value was high, an important characteristic of models used for population-based screening. While an AUC of 0.67 is generally considered moderate performance, it is also comparable to several tests that are widely used in clinical practice.

**Supplementary Information:**

The online version contains supplementary material available at 10.1186/s12911-022-01864-z.

## Background

The U.S. population is aging and age-related diseases like Alzheimer’s Disease and related dementias (ADRD) are becoming more prevalent [1, 2]. ADRD are brain disorders that cause problems with memory, thinking and behavior [3]. Symptoms usually develop slowly and get worse over time and there is no known cure [3]. “Mild cognitive impairment” (MCI) is a defined as cognitive decline greater than expected for an individual’s age and educational attainment [4]. MCI is often diagnosed during the symptomatic predementia phase of ADRD [4]. Subsequent to patient- and family-reported symptoms (e.g., memory deficits), primary care clinicians sometimes administer a standardized screening instrument such as the Mini Mental State Exam (MMSE) [5] or Montreal Cognitive Assessment (MoCA) [6] to identify MCI. However, there is currently insufficient evidence to support universal screening with these instruments [7]. Thus, screening is not performed routinely and as much as half of cognitive impairment goes unrecognized and undiagnosed in primary care [8]. Thus, routine use of machine learning methods applied to clinical notes could speed the time to identification and case management of MCI—thereby enabling earlier psychosocial intervention and reduction in the disease burden [9, 10] and reducing the delay of skills training for home-based care providers (spouses and adult children) who often need training on better coping strategies [11]. Early intervention can also be cost effective [10].

A variety of approaches have been explored to better detect cognitive impairment including identifying patterns of health care utilization prior to diagnosis [12], use of audio recordings to complement neurocognitive testing [13], and analysis of transcript data to identify changes in cognition over time [14]. Ford and coauthors provide a nice review of how structured and unstructured data from primary care electronic health records can be used to predict dementia [15]. Most research into predicting changes in cognition has focused on the use of structured data such as medication utilization [16–22], diagnoses [23–29], procedures [30], and social determinants of health [31–36] that are associated with developing full-blown Alzheimer’s Disease or related dementias (ADRD) [15, 37–43]. We are not aware of any published studies that have tested models predicting development of mild cognitive impairment (MCI) or data abstracted from clinical notes using natural language processing to predict MCI or ADRD, though, Sanghavi and Noderer are conducting work in this area [44]. Berisha et al. [14] discovered declines in language complexity with the progression of Alzheimer’s Disease using transcript data. Kharrazi and colleagues have also reported that the prevalence of geriatric syndromes is significantly under-estimated using structured data alone and that many geriatric syndromes are likely to be missed if unstructured data (i.e., clinical text) are not analyzed [45]. Dementia was one characteristic more highly correlated with descriptions of “frailty” in the research on geriatric syndromes [46].

The purpose of this study was to develop and evaluate a machine learning model employing predictors derived from a natural language processing (NLP) system for identifying patients with MCI from routinely collected clinical notes in patients’ electronic health record.

## Methods

### Overview

There were 4 main steps in our approach to developing the prediction model: (1) developing and applying the NLP system; (2) training a classifier in a gold standard population using the output from the NLP system; (3) refinement of training the classifier in a general population of individuals; and (4) validation of the prediction model in a withheld sample of general population individuals.

This study involved two groups of patients: participants in the Adult Changes in Thought study [47] and a general population cohort of patients receiving care at Kaiser Permanente Washington (KPWA) with Mini-mental State Exam (MMSE) scores or Montreal Cognitive Assessment (MoCA) scores. The study period is January 1, 2004 through September 30, 2015.

We first trained an NLP system on the routine clinic notes of 100% of the ACT cohort participants (n = 1473) and 60% (n = 1435) of the general population cohort to classify patients as positive or negative for symptoms and complaints associated with MCI. We subsequently trained a classifier to predict MCI as independently measured by the MMSE or MoCA score. We used a threshold score of 26 for both the MMSE and MoCA to identify a positive test [48–50].

Prior to developing the NLP system and training machine learning models we selected a 40% (n = 956) random sample of the general population cohort individuals to withhold for model validation. We validated the classifier using clinical text and scores in this 40% withheld general population sample.

## Population

### General population cohort

Our general population cohort included individuals aged ≥ 65 years with an MMSE or MoCA assessment and who were continuously enrolled for two years prior to administration of the assessment. During the study period, 9.7% (n = 15,396/158,937) of individuals in the general population meeting inclusion for the study had MMSE or MoCAs administered due to concerns about memory (and not part of a screening program).

Subjects from the general population were excluded if there was evidence of a diagnosis of mild cognitive impairment, Alzheimer’s Disease or related dementia, Parkinson’s Disease, or psychotic disorder, and/or use of a medication to treat Alzheimer’s Disease (e.g., donepezil) in the clinical record in the 2 years prior to the MMSE or MoCA assessment.

### ACT cohort

The Adult Changes in Thought (ACT) study [47] includes randomly selected, cognitively intact KPWA members. Participants were required to be 65 years of age or older at the time of enrollment, which occurred from 1994 through 1996. A similar group of participants was enrolled between 2000 and 2002. Participants were invited to return at 2-year intervals to identify incident cases of dementia [47].

ACT study participants were assessed for dementia at baseline and every 2 years thereafter by the Cognitive Abilities Screening Instrument (CASI), with scores ranging from 0 to 100 where higher scores indicate better cognitive functioning [51–53]. We translated CASI scores to MMSE scores using a validated crosswalk previously developed in ACT [47]. Dementia-free participants continue with scheduled follow-up visits. The *index date* for dementia is recorded as the midpoint between the study visit when dementia was first diagnosed and the previous study visit [47, 53].

We selected a subset of ACT participants who were continuously enrolled in KPWA for 2 years prior to their index date so they would have, in addition to ACT study-specific data, electronic encounter notes from routine care required for the NLP system. The *index date* for individuals in the ACT cohort was defined by the first *positive* CASI score (score ≤ 85, indicating mild cognitive impairment).

## Data

### Adult changes in thought (ACT) data

Data on ACT participants (diagnoses, CASI test scores, dates of exams) were obtained from the ACT data repository maintained at the Kaiser Permanente Washington Health Research Institute.

### Health system data and virtual data warehouse

Information on enrollment and health care utilization including diagnoses, procedures, and pharmacy dispensings, are recorded and maintained at KPWA in a virtual data warehouse (VDW) [54].

### Developing and applying the NLP system

Developing and applying our NLP system involved: (1) assembling clinical notes for processing, (2) identifying MCI-related concepts, (3) annotating clinical notes, and (4) extracting relevant information from clinical notes to include in the prediction model.

### Assembling clinical notes for processing

Clarity® is the relational database for data extracted from the Epic® EHR. It contains structured EHR data and free-text clinical “notes”. A “note” is the free text section of documentation for a clinical encounter recorded in an electronic health record. Clinicians may enter information about socio-demographic context, impressions of the patient, patient history, or supporting information for a diagnosis (e.g., symptoms/complaints). Notes vary in length between a few characters and several hundred words and may contain information copied and pasted from elsewhere in a patient’s EHR. In addition to the presence of characteristics/features, clinicians may also document the absence of these characteristics/features (e.g., “patient denies problems with sleep”). The notes used in this study are the routinely collected notes in the Kaiser Permanente Washington health system and are broadly representative of documentation found across Kaiser Permanente systems and other health care organizations.

For NLP system training and analyses we used all Family Practice (Primary Care) and Behavioral Health encounter notes during the two years preceding a patient’s index date if that date occurred between January 1, 2004 and September 30, 2015. We chose the study period start date based on availability of encounter notes for ACT enrollees. We limited our corpus of notes to those from the departments of Family Practice and Behavioral Health because these are the settings in which patients are most likely to report cognitive issues to their physician. We excluded Neurology and Speech and Language Pathology notes because they are settings where known cognitive deficits are likely to be referred for follow-up. We were interested in identifying patients that had similar complaints or deficits but did not appear to have appropriate follow-up. Separate corpora were constructed for ACT patients and general population patients.

We defined an index date as the first occurrence of a structured diagnosis for MCI in a patient’s electronic health record. Patients who never received a diagnosis of MCI were matched 1:1 by age, sex, race/ethnicity, and occurrence of a health care visit during the same 3-month calendar period to those who did receive an MCI diagnosis. That is, control cases inherit their index date from their matched MCI cases. The corpora included all notes in the 730 days preceding the index date.

The goal of the NLP investigation was to identify people with evidence of MCI noted in free text that was not recorded/documented in structured diagnosis or pharmacy data in the 2 years prior to the index date. We classified people as positive or negative for evidence of MCI and used this information as an input to predict future MCI status as independently measured by MMSE or MoCA scores in the medical record (i.e., structured data) on the index date.

### Identifying MCI-related concepts and annotation

The first step in building an NLP system was to identify relevant terms and phrases which might indicate MCI. We manually reviewed notes from the ACT cohort to identify an initial set of terms and phrases. These were expanded through further manual review of notes sampled from the general population corpus and loaded into a chart abstraction interface called brat [55]. Three abstractors (TD, AG, RP) reviewed 10,391 notes and highlighted sections of text which might indicate MCI. These results were reviewed, and the most significant terms and phrases were grouped semantically into 42 unique concepts (CUIs) which are presented along with a brief description in Table 1. Linguistically equivalent word form variations were added (“call” → “called”, “calling”, “calls”). The complete list of terms and phrases used along with the associated CUIs is included as an Additional file 1: Appendix. The rules for identifying text are also included as an Additional file 2: Appendix.

**Table 1:** Concepts associated with mild cognitive impairment

Variable	Description	ACT count	Gen. Pop. Pop. count
S_EXCL	References to stroke (used to exclude patients from analysis)	351,995	21,423
WITHX	Patient accompanied by family member	212,669	14,603
RESPONS	Responsibility being assumed by family member	123,834	19,895
NEGATE	Atenolol, hypercalcemia, statins, and “remember to take” boilerplate language	122,559	8703
HALLUC	Hallucination issues	75,042	6515
HEADACHE	Headache/concern for stroke or brain injury	67,955	5550
W_EXCL	Traumatic brain injury, dehydration, etc. (used to exclude patients from analysis)	49,853	3091
DECLINE	Declining memory/cognitive abilities	49,522	13,559
WANDER	Wandering, getting lost, or unable to recognize	42,677	1821
CALLED	Reference to communication going through family member	36,073	3140
FORGET	Forget/can't remember	28,412	3310
DONEPEZIL	Donepezil, Aricept discussed (e.g., regarding what the medications can do)	18,910	821
CONCERN	Family showing concern for patient	15,576	3568
FORGETFL	Forgetful	11,146	1372
EXAM	Cognitive evaluation	10,828	6027
OTHER_SA	Communication goes through family members	5474	562
S_HALLUC	Strong hallucination concern	4791	294
ICD_EXCL	Dementia ICD diagnosis code appearing in text	3622	60
DEMENTIA	Severe dementia noted	2426	85
REFERAL	Referral for cognitive assessment	1653	659
COMPREHE	Poor understanding/comprehension	1606	184
W_DECLIN	Decline in word finding, vocabulary, explaining, etc	1548	148
CONCENTR	Difficulty concentrating	1363	545
EARLY	Early dementia	1204	344
DECLINE_	Communication/call concerning memory decline	1127	87
FORGETX	Forget [something] e.g., keys	978	115
S_CONCER	Worsening or strong concern for dementia	861	237
PLAN	Related care plan to family member	829	47
HAL_EXCL	Hallucination issues resolved	742	79
BOI_INCL	Boilerplate text describing memory problems not necessarily specific to the patient	559	141
OTH_EXCL	Headache/memory complaint relating to non-patient	409	22
RISK	Risk of dementia	379	139
W_CONCER	Concern for word finding, vocabulary, explaining, etc	377	26
ICD_INCL	MCI ICD diagnosis code appearing in text but not in structured data	255	82
DENIAL	Patient denies problem with memory or functioning	204	6
EXM_EXCL	Normal cognitive exam	137	203
STIMULANT	Stimulant medications (modafinil, Provigil, etc.)	113	37
SENILE	Not thinking well/not lucid	73	0
BURDEN	Burden on family member	58	15
BOOK	Names of relevant books, including 36-h Day, Dignified Life, and Ageless Outings	41	1
EXCLUDE	Words referencing forgetfulness excluded because of ambiguity concerns	3	7
WELLNESS	Wellness check	0	0

### Extracting relevant information from clinical notes

Using a locally developed Python program called pyTAKES [56–58] we extracted terms and phrases from notes corresponding to each concept (Table 1) in both the ACT cohort and general population cohort. pyTAKES identifies the terms and phrases from the list by first isolating sentences from the input note and tokenizing each sentence. pyTAKES then examines the tokenized input to determine if the target term matches any token. When searching for a phrase (e.g., the CUI “DECLINE” is associated with the phrase “loss cognitive ability”), pyTAKES looks for each word in succession, allowing for up to two intervening words. For example, “loss cognitive ability” will match “loss of cognitive ability”. The immediate contexts of each term (i.e., the 180 characters immediately before and after) are also retained allowing for a subsequent step to remove boilerplate (i.e., template language). Boilerplate was eliminated by identifying terms that shared either the same previous 180 characters or subsequent 180 characters with other patients.

All of the identified concepts were then supplied as features to the predictive model as binary features: coded 1 if any CUI was present in the patient’s notes, and 0 otherwise.

#### Machine learning model inputs

The NLP system described above identified people with documentation of symptoms and complaints of MCI but who did not have a diagnosis or treatment for MCI or dementia at the time the clinical note was entered. The next step in building the predictive model was to expand our pool of potential predictors available to our prediction model. We included imputed household income and imputed education from census data based on where patients lived, as well as patient demographic information in the form of age, sex, and race/ethnicity. Additionally, based on clinical judgement, we specified three aggregate predictors from the concepts in Table 1. There was one aggregate predictor for symptoms, one for behaviors, and one for forgetfulness. We calculated each as the sum of occurrences of relevant CUIs in a patient’s notes as follows: Symptom Sum = (WANDER + FORGET + FORGETFL + CONCENTR + DECLINE + W_DECLIN + COMPREHE + S_HALLUC + RISK); Behavior Sum = (CONCERN + CALLED + WITHX + S_CONCER + W_CONCER + REFERAL + PLAN); and Forgetful Sum = (FORGET + FORGETFUL + FORGETX). Thus, the Symptom Sum varies between 0 and 9, the Behavior Sum varies between 0 and 7, and the Forgetful Sum varies between 0 and 3). Please refer to Table 1 for the definitions of the concepts [59–62].

### Machine learning statistical approach

We used a least absolute shrinkage and selection operator (LASSO) logistic regression approach [63] to construct a prediction model on our general population training dataset using the NLP-derived concepts and demographic variables. The LASSO approach retains the subset of predictors with the strongest effects by shrinking some coefficients to zero and thereby improves model interpretability [64]. We used tenfold cross-validation to estimate the tuning parameter. The optimal amount of shrinkage was established using ten-fold cross-validation.

Our prediction target was a binary indicator of MCI present/absent based on a MoCA or MMSE score > 26 or ≤ 26 on the index date. Predictor variables included patient age, sex, and race, presence or absence of each of the concepts we identified, and each of the three symptom scores. Using the concepts identified from the NLP system and known MCI from MMSE or MoCA scores, we constructed receiver operating curves (ROC) to measure the performance of the LASSO model in correctly predicting MCI status. We specified a range of cutoff points and performance characteristics (sensitivity, specificity, PPV, NPV) were evaluated on both training and validation datasets.

This project was approved by the Kaiser Permanente Washington institutional review board.

## Results

### Corpus

There were 143,153 notes for 1473 ACT patients and 23,579 notes for 2391 general population patients. Table 2 shows the characteristics of the notes across corpora. Overall there were 1365,406 unique occurrences of the 42 concepts. The most frequently mentioned concepts were S_EXCL (exclude based on stroke noted, n = 373,418), WITHX (patient is accompanied by a loved one, n = 227,272), RESPONS (language noting a family member is taking responsibility for the care plan, n = 143,729) and NEGATE (clinician advising patient not to forget to do something such as take their hypertension medication, n = 131,262). Concepts affirmatively characterizing behaviors or symptoms of MCI were less common.

**Table 2:** Corpora descriptive statistics for characters, words, and tokens

Corpus	Num. chars	Num. chars	Num. chars	Num. words	Num. words	Num. words	Num. tokens	Num. of tokens	Num. tokens
	Mean	Max	Min	Mean	Max	Min	Mean	Max	Min
ACT (training)	1229.6	52,491.0	0	216.9	9350.0	0	260.8	10,946.0	0
Gen. Pop. (training)	1324.9	76,831.0	0	233.0	15,029.0	0	276.9	17,422.0	0
Gen. Pop. (validation)	1118.7	58,080.0	0	196.9	9588.0	0	234.9	11,251.0	0

### Population

Table 3 shows the demographic characteristics of the ACT cohort and general population cohort. We initially identified 15,396 people in the general population that were aged 65 years or more with an MMSE or MoCA score. Of these, 2071 were excluded because they were not continuously enrolled for 2 years prior to the index date on which the instrument was completed. Of the remaining 13,325 individuals, 5979 were excluded for a diagnosis of ADRD, 938 for a diagnosis of psychosis, 693 for a diagnosis of MCI, and 1739 for bipolar disorder. Of the remaining 6858, a further 488 were excluded for antipsychotic medication use and 386 who were enrolled in the ACT study. Finally, 711 were excluded because they had no notes with clinical text in the two years prior to their index test producing a final general population cohort of 2391.

**Table 3:** Cohort demographics

	ACT cohort		General population	
	N	%	N	%
Total people	1473	100	2391	100
Age at index				
65–69	14	0.95	456	19.07
70–74	107	7.26	515	21.54
75–79	260	17.65	461	19.28
80–84	395	26.82	450	18.82
85+	697	47.32	509	21.29
Sex				
Female	954	64.77	1419	59.35
Male	519	35.23	972	40.65
Race				
American Indian/Alaska native	8	0.54	26	1.09
Asian	40	2.72	104	4.35
Black or African American	61	4.14	51	2.13
Native Hawaiian or Other Pacific Islander	3	0.2	1	0.04
Other	10	0.68	15	0.63
Unknown or not reported	27	1.83	46	1.92
White	1324	89.88	2148	89.84
Ethnicity				
Hispanic or Latino	37	2.51	94	3.93
Not Hispanic or Latino	1417	96.2	2249	94.06
Unknown/not reported ethnicity	19	1.29	48	2.01
Neighborhood income				
< $25,000	6	0.41	21	0.88
≥ $25,000	1409	95.66	2351	98.33
Missing	58	3.94	19	0.79
Neighborhood education				
< 25% college	209	14.19	795	33.25
≥ 25% college	1206	81.87	1577	65.96
Missing	58	3.94	19	0.79

The prevalence of MCI (as measured by test scores) varied across the cohorts. In the ACT training data, the prevalence of MCI was 50.03%. In the general population training data, the prevalence of MCI was 42.9% and in the general population validation data set the prevalence was 29.8%.

Table 4 shows the observed prevalence of MCI by age group and sex in the ACT cohort and General Population cohorts.

**Table 4:** MCI prevalence

		ACT cohort		General population	
		MCI (−)	MCI (+)	MCI (−)	MCI (+)
Age					
65–69	n	9	5	371	85
	%	64.3	35.7	81.4	18.6
70–74	n	57	51	407	108
	%	52.8	47.2	79.0	21.0
75–79	n	136	123	318	143
	%	52.5	47.5	69.0	31.0
80–84	n	194	200	270	180
	%	49.2	50.8	60.0	40.0
85 +	n	341	357	283	226
	%	48.9	51.1	55.6	44.4
Sex					
Male	n	260	259	693	279
	%	50.1	49.9	71.3	28.7
Female	n	477	477	956	463
	%	50.0	50.0	67.4	32.6
Total	n	737	736	1649	742
	%	50.0	50.0	69.0	31.0

Table 5 shows the results of the logistic LASSO model. Age is a well-known predictor of cognitive impairment and this is borne out in the current study. With a coefficient of 0.023 per year, the coefficient for an individual aged 70 years would be 1.61. Stated another way, 8 years of aging is about the same in terms of MCI risk as documentation of communication going through family members.

**Table 5:** Variables retained in the prediction model

Variable (intercept)	Description	Coefficient
ICD_EXCL	Dementia ICD9 codes in text but not structured data	0.634
DEMENTIA	Severe dementia	0.596
DONEPEZIL	Discussion of Aricept, donepezil (but not prescribed or used)	0.568
OTHER_SA	Communication goes through family members	0.17
RACE	Black race	0.134
DECLINE	Declining memory/cognitive abilities	0.082
BEHAVIOR SUM	Sum of presence of behavioral concepts	0.076
AGE	Age at index (per year) e.g. For age 70 the coefficient = 1.61	0.023
CALLED	Reference to family member calling about patient’s memory	0.012

Of the concepts identified in encounter notes, mention of donepezil, text indicating severe dementia, and problem list codes for dementia in text (but not structured data) were the strongest coefficients. With a coefficient of 0.134, Black race was also a significant predictor of MCI. On the other hand, variables such as communication through family members, and declining cognitive abilities had relatively weak coefficients. Concepts such as wandering, and hallucinations were not retained by the model.

**Fig. 1:**
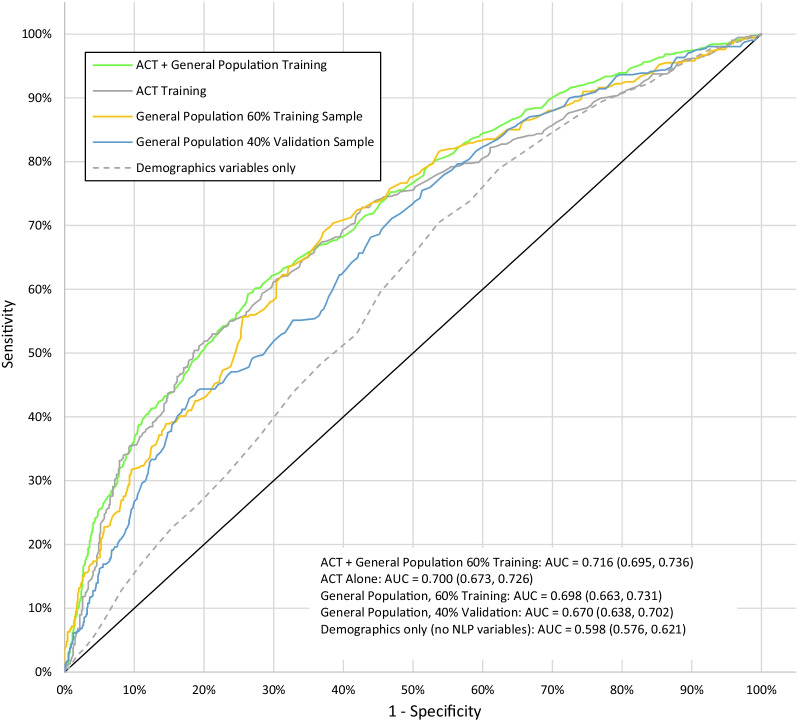
ROC curve for training and validation cohorts. Green dotted line: ACT + general population training. Light green dotted line: ACT training. Orange dotted line: general population 60% training sample. Blue dotted line: general population 40% validation sample. Gray dotted line: demographic variables only. ACT + general population 60% training: AUC = 0.716 (0.695, 0.736). ACT alone: AUC = 0.700 (0.673, 0.726). General population, 60% Training: AUC = 0.698 (0.663, 0.731). General population, 40% validation: AUC = 0.670 (0.638, 0.702). Demographics only (no NLP variables): AUC = 0.598 (0.576, 0.621)

Figure 1 shows the ROC curve characterizing performance of the model created using logistic LASSO. The area under the curve (AUC) for the validation data set is 0.67. Sensitivity analyses using only demographic variables produced an AUC of 0.598 suggesting that the NLP-derived variables significantly improve predictive ability over demographics alone. Because there is always a trade-off between sensitivity and specificity, Table 6 presents sensitivity, specificity, PPV, NPV across a wide range of cut-points (corresponding to different probabilities of correct classification). The prediction model generates a probability of MCI present at index date (which ranges from 0 to 1). For example, a cutoff of 0.3 corresponds to a 30% predicted probability of MCI diagnosed at index date. Setting the cutoff for correct classification in the general population validation cohort to 0.60 yields sensitivity of 0.02, specificity of 1.0, PPV of 0.70, NPV of 0.70 and F1 score of 0.04.

**Table 6:** Prediction model performance characteristics in each population at various cutoffs for probability of correct classification

Cohort	Cutoff^a^	Sensitivity	Specificity	PPV	NPV	F1 Score
ACT + Gen. Pop. training	0.3	0.95	0.17	0.46	0.83	0.62
	0.4	0.63	0.69	0.6	0.71	0.61
	0.5	0.37	0.9	0.73	0.66	0.49
	0.6	0.24	0.96	0.81	0.63	0.37
ACT training	0.3	0.99	0.04	0.51	0.76	0.67
	0.4	0.75	0.52	0.61	0.68	0.67
	0.5	0.49	0.82	0.73	0.62	0.59
	0.6	0.34	0.92	0.80	0.58	0.48
Gen. Pop. training	0.3	0.88	0.31	0.38	0.84	0.53
	0.4	0.37	0.86	0.57	0.74	0.45
	0.5	0.11	0.98	0.75	0.70	0.19
	0.6	0.02	1.00	1.00	0.68	0.04
Gen. Pop. validation	0.3	0.87	0.32	0.35	0.85	0.50
	0.4	0.32	0.88	0.53	0.75	0.40
	0.5	0.09	0.97	0.56	0.71	0.16
	0.6	0.02	1.00	0.70	0.70	0.04

^a^Cutoffs are the various probabilities that the researcher or health system would choose as a threshold to classify someone as “positive” for MCI

## Discussion

Several studies report increased health care utilization and costs of care prior to diagnosis of Alzheimer’s Disease or Dementia [65–71]. While the largest increases appear to occur in the 3–6 months prior to diagnosis [66], other studies report significant increases in utilization in the 1–3 years prior. Our study focused on the identification of mild cognitive impairment (rather than Alzheimer’s or Dementia) in the absence of screening to identify individuals on a trajectory of cognitive decline as early as possible. Early identification may help focus health care resources because identifying individuals as early as possible enables clinicians to offer patients education about the disease process and caregiver support interventions that reduce the burden of disease. Early identification also gives patients time to complete advanced directives and other end of life planning while they are still cognitively capable of doing so.

### General performance of the prediction model

Among the 42 concepts identified by clinical experience and manual chart review, concepts that negated or ruled-out cognitive impairment were documented more frequently than those that positively identified individuals. The more common documentation of negating concepts is reflected in the very high specificity of the model across cohorts. It is also notable that the total amount of EHR information available (driven by the number of EHR notes available) was much greater for ACT than general population patients. One possible reason for the relatively modest performance of the prediction model in the validation data set may be that individuals in the general population had fewer contacts with the health system and therefore had less documentation in the EHR. It is well known that the stage of first presentation of cognitive decline varies greatly among individuals. Some patients seek care (or divulge cognitive issues) when symptoms are quite mild while others seek care only after symptoms are severe. Patients in the ACT cohort were assessed every 6 months for cognitive function and were cognitively intact at baseline according to inclusion criteria. Our approach is only useful for early detection and intervention insofar as people make health care visits and documentation of mild symptoms exists in the HER—especially in the absence of regular, standardized screening. Previous studies have reported a bolus of health care utilization in the months leading up to an Alzheimer’s Disease or dementia diagnosis [66, 69] but not a diagnosis of mild cognitive impairment.

While an AUC of 0.67 is generally considered moderate test performance, it is also comparable to several tests that are widely used in clinical practice. For example, Veltri and Miller [72] reported an AUC of 0.632 for total prostate specific antigen (tPSA) in differentiating benign from malignant prostate tumors in a sample of 4870 patients. Similarly, Flueckiger and colleagues [73] reported an AUC of 0.716 for the revised Framingham Stroke Risk Score. The AUC for the Papanicolaou smear in detecting cervical intraepithelial neoplasia is 0.689 [74].

### Concepts retained by the prediction model

It is well known that risk of cognitive impairment increases with age and this is reflected in the magnitude of the coefficient for age in the LASSO model. Adjusting for age, the concepts mostly strongly associated with positive MoCA and MMSE scores were related to more severe cognitive deficits. These included mentions of (but not the use of) donepezil, severe cognitive decline, and free text diagnosis codes from the problem list (but not included as formal diagnoses). This suggests that the model performs better when patients have more advanced cognitive impairment at assessment and therefore more documentation of symptoms and complaints. This may happen when patients wait to seek help until their functioning is significantly impacted. The utility of our approach in detecting cognitive impairment early depends on patients making visits and clinicians documenting mild symptoms.

The second general class of concepts retained in the model are related to communication and/or concern by family members about the patient. Both the occurrence of such communication and the cumulative amount of this communication (as measured by the aggregate variable Behavior Sum) were retained as significant predictors. This result is interesting from a clinical intervention perspective because signals/alerts for follow-up could be generated for case managers or physicians when the volume of communication by family members about the patient increases (both about memory and physical conditions).

The final interesting result is the retention of black race in the prediction model. Like the variables retained for evidence of more severe cognitive impairment discussed above, one interpretation of this result is that African American individuals are less likely to have their cognitive status discussed during an office visit until the disease has progressed to significant impairment. It may also be true that MoCA or MMSE measurement of African Americans may tend to be delayed until significant impairment exists relative to measurement of individuals of other races.

### Limitations

This study has some important limitations. First, we conducted the study in one health system and the documentation of symptoms and complaints is likely to differ across health systems. Thus, the performance of the prediction model may be better or worse if these analyses were replicated elsewhere. Second, the training of the prediction model to ACT participants was both a strength and weakness. Research participants may not be representative in terms of visit frequency, education, and other characteristics. On the other hand, we leveraged ACT patients’ CASI scores and known periods of intact cognitive status to train the prediction model. We observed that ACT participants had significantly more contact with the health system (separate from their participation in research) and thus more documentation with which to train the model than was available in the general population.

Third, we did not evaluate the performance of the NLP system in correctly identifying concepts contained in notes. This would require comparing the automated identification in the study corpus to a reference corpus which we did not have or create. Instead, we focused on comparing our automated identification to the standardized measures of cognitive function. Similarly, we did not conduct inter-rater reliability analysis of notes that were manually annotated for concept discovery.

Fourth, there is potential for measurement error and bias in the general population of individuals with MoCA or MMSE scores. These screening instruments are not equivalent and have different performance characteristics. Moreover, a positive screen is not sufficient to diagnose mild cognitive impairment (though positive scores are routinely used to give diagnoses and refer patients to specialty care). Also, these instruments are administered when clinicians suspect cognitive impairment or want to rule-out impairment when patients or family members report symptoms, as opposed to being used for universal screening. While this bias exists, it is unlikely to have affected our results significantly because 69.0% of the MoCA and MMSE scores in the general population were negative. Also, the specificity of the model was much higher than the sensitivity. Measurement bias (by selective administration) would be more worrisome if the sensitivity of the model were very high. It is also worth noting that the prevalence of cognitive impairment increases with age. A test with the same sensitivity and specificity administered in a population with a higher prevalence will produce a higher positive predictive value and lower negative predictive value [75]. The prevalence of MCI in our training data set was intentionally set to 50% (by matching); however, the prevalence of MCI in the tested general population was only 31%.

Finally, we only calibrated one prediction model using a LASSO approach. It is possible that a different approach (such as a random forest model or neural network model) would perform better. We did not pursue these other models (and compare performance) for two reasons. First, the computational resources needed to estimate the more complicated models greatly exceed those available to healthcare systems and clinics who would use these predictive models. Second, LASSO models can be implemented natively in many electronic health records (EHR). This capability enables prediction models to be updated natively within the EHR as new healthcare utilization data become available. We are interested in ML approaches that can be implemented in the real world and change clinical care.

## Conclusion

We were able to identify concepts appearing in clinical notes that are predictive of individuals developing mild cognitive impairment at a future date. The model performs moderately well in predicting MCI; however, performance may be improved by including covariates identified here with structured data in the medical record such as other diagnoses, injuries (e.g., falls), and patterns of utilization (e.g., increases in primary care visits). The success of future work on predictive modeling of cognitive impairment is likely to depend on a machine learning approach that incorporates multiple sources of data and discovering previously unidentified features.

## Supplementary Information

**Additional file 1:** NLP Dictionary.

**Additional file 2:** NLP Rule Definitions for Concept Unique Identifiers.

## Data Availability

Data supporting the results reported in this article may be obtained upon request from the corresponding author. Please email robert.b.penfold@kp.org to request data. Because these data could compromise individual privacy, a signed data use agreement will be necessary to access the analytic file. We cannot make available the clinical free text from patient electronic health records.

## Abbreviations

ACT: Adult Changes in Thought study
ADRD: Alzheimer's Disease and Related Dementias
AUC: Area under the curve
BRAT: Brat rapid annotation tool
CASI: Cognitive Abilities Screening Instrument
CUI: Concept unique identifier
EHR: Electronic health record
Gen. Pop.: General population
KPWA: Kaiser Permanente Washington
LASSO: Least absolute shrinkage and selection operator
MCI: Mild cognitive impairment
MMSE: Mini Mental State Exam
MoCA: Montreal Cognitive Assessment
NLP: Natural language processing
NPV: Negative predictive value
PPV: Positive predictive value
ROC: Receiver operating curve
U.S.: United States
VDW: Virtual data warehouse

## References

[1] Plassman BL, Langa KM, Fisher GG, Heeringa SG, Weir DR, Ofstedal MB, Burke JR, Hurd MD, Potter GG, Rodgers WL (2007). Prevalence of dementia in the United States: the aging, demographics, and memory study. Neuroepidemiology.

[2] International AsD (2009). World Alzheimer Report 2009: the global prevalence of dementia.

[3] What is Alzheimer's. https://www.alz.org/alzheimers-dementia/what-is-alzheimers.

[4] Gauthier S, Reisberg B, Zaudig M, Petersen RC, Ritchie K, Broich K, Belleville S, Brodaty H, Bennett D, Chertkow H (2006). Mild cognitive impairment. The Lancet.

[5] Folstein MF, Folstein SE, McHugh PR (1975). Mini-mental state: a practical method for grading the cognitive state of patients for the clinician. J Psychiatr Res.

[6] Nasreddine ZS, Phillips NA, Bedirian V, Charbonneau S, Whitehead V, Collin I, Cummings JL, Chertkow H (2005). The Montreal Cognitive Assessment, MoCA: a brief screening tool for mild cognitive impairment. J Am Geriatr Soc.

[7] Moyer VA (2014). (2014) Force USPST: screening for cognitive impairment in older adults: US Preventive Services Task Force recommendation statement. Ann Intern Med.

[8] Amjad H, Roth DL, Sheehan OC, Lyketsos CG, Wolff JL, Samus QM (2018). Underdiagnosis of dementia: an observational study of patterns in diagnosis and awareness in US older adults. J Gen Intern Med.

[9] Herman L, Atri A, Salloway S (2017). Alzheimer's Disease in primary care: the significance of early detection, diagnosis, and intervention. Am J Med.

[10] Barnett JH, Lewis L, Blackwell AD, Taylor M (2014). Early intervention in Alzheimer's disease: a health economic study of the effects of diagnostic timing. BMC Neurol.

[11] McNair T (2015). Early intervention for caregivers of patients with Alzheimer's Disease. Home Healthc Now.

[12] Nair R, Haynes VS, Siadaty M, Patel NC, Fleisher AS, Van Amerongen D, Witte MM, Downing AM, Fernandez LAH, Saundankar V (2018). Retrospective assessment of patient characteristics and healthcare costs prior to a diagnosis of Alzheimer's disease in an administrative claims database. BMC Geriatr.

[13] Roark B, Mitchell M, Hosom JP, Hollingshead K, Kaye J (2011). Spoken language derived measures for detecting mild cognitive impairment. IEEE Trans Audio Speech Lang Process.

[14] Berisha V, Wang S, LaCross A, Liss J (2015). Tracking discourse complexity preceding Alzheimer's disease diagnosis: a case study comparing the press conferences of Presidents Ronald Reagan and George Herbert Walker Bush. J Alzheimers Dis.

[15] Ford E, Greenslade N, Paudyal P, Bremner S, Smith HE, Banerjee S, Sadhwani S, Rooney P, Oliver S, Cassell J (2018). Predicting dementia from primary care records: a systematic review and meta-analysis. PLoS ONE.

[16] Breitner JC, Haneuse SJ, Walker R, Dublin S, Crane PK, Gray SL, Larson EB (2009). Risk of dementia and AD with prior exposure to NSAIDs in an elderly community-based cohort. Neurology.

[17] Dublin S, Walker RL, Gray SL, Hubbard RA, Anderson ML, Yu O, Crane PK, Larson EB (2015). Prescription opioids and risk of dementia or cognitive decline: a prospective cohort study. J Am Geriatr Soc.

[18] Gray SL, Anderson ML, Dublin S, Hanlon JT, Hubbard R, Walker R, Yu O, Crane PK, Larson EB (2015). Cumulative use of strong anticholinergics and incident dementia: a prospective cohort study. JAMA Intern Med.

[19] Gray SL, Dublin S, Yu O, Walker R, Anderson M, Hubbard RA, Crane PK, Larson EB (2016). Benzodiazepine use and risk of incident dementia or cognitive decline: prospective population based study. BMJ.

[20] Gray SL, Walker RL, Dublin S, Yu O, Aiello Bowles EJ, Anderson ML, Crane PK, Larson EB (2018). Proton pump inhibitor use and dementia risk: prospective population-based study. J Am Geriatr Soc.

[21] Helmstaedter C, Beghi E, Elger CE, Kalviainen R, Malmgren K, May TW, Perucca E, Trinka E (2018). No proof of a causal relationship between antiepileptic drug treatment and incidence of dementia—Comment on: use of antiepileptic drugs and dementia risk—an analysis of Finnish health register and German health insurance data. Epilepsia.

[22] Hwang D, Kim S, Choi H, Oh IH, Kim BS, Choi HR, Kim SY, Won CW (2016). Calcium-channel blockers and dementia risk in older Adults- National Health Insurance Service - Senior Cohort (2002–2013). Circ J.

[23] Bos I, Vos SJ, Frolich L, Kornhuber J, Wiltfang J, Maier W, Peters O, Ruther E, Engelborghs S, Niemantsverdriet E (2017). The frequency and influence of dementia risk factors in prodromal Alzheimer's disease. Neurobiol Aging.

[24] Cherbuin N, Kim S, Anstey KJ (2015). Dementia risk estimates associated with measures of depression: a systematic review and meta-analysis. BMJ Open.

[25] Exalto LG, Biessels GJ, Karter AJ, Huang ES, Katon WJ, Minkoff JR, Whitmer RA (2013). Risk score for prediction of 10 year dementia risk in individuals with type 2 diabetes: a cohort study. Lancet Diabetes Endocrinol.

[26] Hessler JB, Ander KH, Bronner M, Etgen T, Forstl H, Poppert H, Sander D, Bickel H (2016). Predicting dementia in primary care patients with a cardiovascular health metric: a prospective population-based study. BMC Neurol.

[27] Martins RN, Gandy S (2016). Prostate cancer: Increased dementia risk following androgen deprivation therapy?. Nat Rev Urol.

[28] Riby LM, Riby DM (2014). Raised blood glucose as a predictor of dementia risk in adults with and without diabetes. Evid Based Med.

[29] Autoimmune disease linked with increased dementia ris. Nurs Stand. 2017; 31:30–17. 10.7748/ns.31.30.17.s20 28327041

[30] Aiello Bowles EJ, Larson EB, Pong RP, Walker RL, Anderson ML, Yu O, Gray SL, Crane PK, Dublin S (2016). Anesthesia exposure and risk of dementia and Alzheimer's Disease: a prospective study. J Am Geriatr Soc.

[31] Defrancesco M (2016). Mediterranean diet and treating diabetes and depression in old age may reduce dementia risk. Evid Based Ment Health.

[32] Hackett RA, Davies-Kershaw H, Cadar D, Orrell M, Steptoe A (2018). Walking speed, cognitive function, and dementia risk in the English longitudinal study of ageing. J Am Geriatr Soc.

[33] Harada K, Lee S, Lee S, Bae S, Anan Y, Harada K, Shimada H (2018). Expectation for physical activity to minimize dementia risk and physical activity level among older adults. J Aging Phys Act.

[34] Spartano NL, Ngandu T (2018). Fitness and dementia risk: further evidence of the heart-brain connection. Neurology.

[35] Mura T, Baramova M, Gabelle A, Artero S, Dartigues JF, Amieva H, Berr C (2017). Predicting dementia using socio-demographic characteristics and the Free and Cued Selective Reminding Test in the general population. Alzheimers Res Ther.

[36] Xu W, Wang H, Wan Y, Tan C, Li J, Tan L, Yu JT (2017). Alcohol consumption and dementia risk: a dose-response meta-analysis of prospective studies. Eur J Epidemiol.

[37] Gothlin M, Eckerstrom M, Rolstad S, Wallin A, Nordlund A (2017). Prognostic accuracy of mild cognitive impairment subtypes at different cut-off levels. Dement Geriatr Cogn Disord.

[38] Hogan DB, Ebly EM (2000). Predicting who will develop dementia in a cohort of Canadian seniors. Can J Neurol Sci.

[39] Jang H, Ye BS, Woo S, Kim SW, Chin J, Choi SH, Jeong JH, Yoon SJ, Yoon B, Park KW (2017). Prediction model of conversion to dementia risk in subjects with amnestic mild cognitive impairment: a longitudinal. Multi-Center Clinic-Based Study. J Alzheimers Dis.

[40] Kaffashian S, Dugravot A, Elbaz A, Shipley MJ, Sabia S, Kivimaki M, Singh-Manoux A (2013). Predicting cognitive decline: a dementia risk score vs the Framingham vascular risk scores. Neurology.

[41] Korolev IO, Symonds LL, Bozoki AC (2016). Alzheimer's Disease neuroimaging i: predicting progression from mild cognitive impairment to alzheimer's dementia using clinical, MRI, and plasma biomarkers via probabilistic pattern classification. PLoS ONE.

[42] Stephan BC, Tang E, Muniz-Terrera G (2016). Composite risk scores for predicting dementia. Curr Opin Psychiatry.

[43] Tang EY, Harrison SL, Errington L, Gordon MF, Visser PJ, Novak G, Dufouil C, Brayne C, Robinson L, Launer LJ (2015). Current developments in dementia risk prediction modelling: an updated systematic review. PLoS ONE.

[44] Uncovering hidden patterns in dementia that might save lives. https://www.optumlabs.com/blog/blog.entry.html/2017/11/03/uncovering_hiddenpa-4WHZ.html.

[45] Kharrazi H, Anzaldi LJ, Hernandez L, Davison A, Boyd CM, Leff B, Kimura J, Weiner JP (2018). The value of unstructured electronic health record data in geriatric syndrome case identification. J Am Geriatr Soc.

[46] Anzaldi LJ, Davison A, Boyd CM, Leff B, Kharrazi H (2017). Comparing clinician descriptions of frailty and geriatric syndromes using electronic health records: a retrospective cohort study. BMC Geriatr.

[47] Kukull WA, Higdon R, Bowen JD, McCormick WC, Teri L, Schellenberg GD, van Belle G, Jolley L, Larson EB (2002). Dementia and Alzheimer disease incidence: a prospective cohort study. Arch Neurol.

[48] Crane PK, Narasimhalu K, Gibbons LE, Mungas DM, Haneuse S, Larson EB, Kuller L, Hall K, van Belle G (2008). Item response theory facilitated cocalibrating cognitive tests and reduced bias in estimated rates of decline. J Clin Epidemiol.

[49] Damian AM, Jacobson SA, Hentz JG, Belden CM, Shill HA, Sabbagh MN, Caviness JN, Adler CH (2011). The Montreal Cognitive Assessment and the mini-mental state examination as screening instruments for cognitive impairment: item analyses and threshold scores. Dement Geriatr Cogn Disord.

[50] Rossetti HC, Lacritz LH, Cullum CM, Weiner MF (2011). Normative data for the Montreal Cognitive Assessment (MoCA) in a population-based sample. Neurology.

[51] Teng EL, Hasegawa K, Homma A, Imai Y, Larson E, Graves A, Sugimoto K, Yamaguchi T, Sasaki H, Chiu D et al. The Cognitive Abilities Screening Instrument (CASI): a practical test for cross-cultural epidemiological studies of dementia. *Int Psychogeriatr* 1994, **6**(1):45–58; discussion 62. 10.1017/s1041610294001602 8054493

[52] McCurry SM, Edland SD, Teri L, Kukull WA, Bowen JD, McCormick WC, Larson EB (1999). The cognitive abilities screening instrument (CASI): data from a cohort of 2524 cognitively intact elderly. Int J Geriatr Psychiatry.

[53] Crane PK, Walker R, Hubbard RA, Li G, Nathan DM, Zheng H, Haneuse S, Craft S, Montine TJ, Kahn SE (2013). Glucose levels and risk of dementia. N Engl J Med.

[54] Ross TR, Ng D, Brown JS, Pardee R, Hornbrook MC, Hart G, Steiner JF. The HMO research network virtual data warehouse: a public data model to support collaboration. In: eGEMs—generating evidence and methods to improve patient outcomes, vol. 2; 2014. 10.13063/2327-9214.1049 PMC4371424 25848584

[55] Stenetorp P, Pyysalo S, Topić G, Ohta T, Ananiadou S, Tsujii J. brat: a Web-based Tool for NLP-assisted text annotation. Avignon: Association for Computational Linguistics; 2012.

[56] Python Language Reference, version 3.6. http://www.python.org .

[57] Carrell DS, Cronkite D, Palmer RE, Saunders K, Gross DE, Masters ET, Hylan TR, Von Korff M (2015). Using natural language processing to identify problem usage of prescription opioids. Int J Med Inform.

[58] dcronkite/pytakes. https://github.com/dcronkite/pytakes.

[59] Pedregosa F, Varoquaux G, Gramfort A, Michel V, Thirion B, Grisel O, Blondel M, Prettenhofer P, Weiss R, Dubourg V (2011). Scikit-learn: machine learning in python. J Mach Learn Res.

[60] Salton G, Buckley C (1988). Term-weighting approaches in automatic text retrieval. Inf Process Manage.

[61] Yamamoto M, Church KW (2001). Using suffix arrays to compute term frequency and document frequency for all substrings in a corpus. Comput Linguist.

[62] Lucini FR, Fogliatto FS, da Silveira GJC, Neyeloff JL, Anzanello MJ (2017). Text mining approach to predict hospital admissions using early medical records from the emergency department. Int J Med Inform.

[63] Friedman J, Hastie T, Tibshirani R (2010). Regularization paths for generalized linear models via coordinate descent. J Stat Softw.

[64] Tibshirani R (1996). Regression shrinkage and selection via the Lasso. J R Stat Soc Ser B Methodol.

[65] Albert SM, Glied S, Andrews H, Stern Y, Mayeux R (2002). Primary care expenditures before the onset of Alzheimer's disease. Neurology.

[66] Chen L, Reed C, Happich M, Nyhuis A, Lenox-Smith A (2014). Health care resource utilisation in primary care prior to and after a diagnosis of Alzheimer's disease: a retrospective, matched case-control study in the United Kingdom. BMC Geriatr.

[67] Eaker ED, Mickel SF, Chyou PH, Mueller-Rizner NJ, Slusser JP (2002). Alzheimer's disease or other dementia and medical care utilization. Ann Epidemiol.

[68] Gaugler JE, Hovater M, Roth DL, Johnston JA, Kane RL, Sarsour K (2013). Analysis of cognitive, functional, health service use, and cost trajectories prior to and following memory loss. J Gerontol B Psychol Sci Soc Sci.

[69] Geldmacher DS, Kirson NY, Birnbaum HG, Eapen S, Kantor E, Cummings AK, Joish VN (2013). Pre-diagnosis excess acute care costs in Alzheimer's patients among a US Medicaid population. Appl Health Econ Health Policy.

[70] Ramakers IH, Visser PJ, Aalten P, Boesten JH, Metsemakers JF, Jolles J, Verhey FR (2007). Symptoms of preclinical dementia in general practice up to five years before dementia diagnosis. Dement Geriatr Cogn Disord.

[71] Suehs BT, Davis CD, Alvir J, van Amerongen D, Pharmd NC, Joshi AV, Faison WE, Shah SN (2013). The clinical and economic burden of newly diagnosed Alzheimer's disease in a medicare advantage population. Am J Alzheimers Dis Other Demen.

[72] Veltri RW, Miller MC (1999). Free/total PSA ratio improves differentiation of benign and malignant disease of the prostate: critical analysis of two different test populations. Urology.

[73] Flueckiger P, Longstreth W, Herrington D, Yeboah J (2018). Revised framingham stroke risk score, nontraditional risk markers, and incident stroke in a multiethnic cohort. Stroke.

[74] Cardenas-Turanzas M, Follen M, Nogueras-Gonzalez GM, Benedet JL, Beck JR, Cantor SB (2008). The accuracy of the Papanicolaou smear in the screening and diagnostic settings. J Low Genit Tract Dis.

[75] Mausner J, Kramer S (1985). Mausner and bahn epidemiology: an introductory text.

