# Supplementary material for: Development of a machine learning model to predict mild cognitive impairment using natural language processing in the absence of screening
Source: BMC Med Inform Decis Mak. 2022 May 12;22:129. doi: 10.1186/s12911-022-01864-z (PMC9097352; doi:10.1186/s12911-022-01864-z)
Supplement: Supplementary file 2 — Additional file 2: NLP Rule Definitions for Concept Unique Identifiers. [file 12911_2022_1864_MOESM2_ESM.docx]

Appendix 2

NLP Rule Definitions for Concept Unique Identifiers

RESPONS==[subject] [responsibility]%%0,2

RESPONS==[responsibility] [subject]%%0,2

RESPONS==[decline] [responsibility]%%0,2

RESPONS==[responsibility] [decline]%%0,2

RESPONS==[responsibility] [help]%%0,2

RESPONS==[help] [responsibility]%%0,2

RESPONS==[simple_neg] [responsibility]%%0,2

RESPONS==[responsibility_solo]%%0,2

CONCERN==[memory] [concern]%%0,2

CONCERN==[concern] [memory]%%0,2

CONCERN==[subject] [concern]%%0,2

CONCERN==[concern] [subject]%%0,2

S_CONCERN==[modifier] [memory] [concern]%%0,2

S_CONCERN==[concern] [modifier] [memory]%%0,2

S_CONCERN==[subject] [modifier] [concern]%%0,2

S_CONCERN==[modifier] [concern] [subject]%%0,2

W_CONCERN==[concern] [submemory]%%0,2

W_CONCERN==[submemory] [concern]%%0,2

DECLINE==[memory] [decline]%%0,2

DECLINE==[decline] [memory]%%0,2

DECLINE==[short_term] [decline]%%0,2

DECLINE==[decline] [short_term]%%0,2

W_DECLINE==[submemory] [decline]%%0,2

W_DECLINE==[decline] [submemory]%%0,2

COMPREHENSION==[decline] [comprehend]%%0,2

REFERAL==[refer] [speech]%%0,2

REFERAL==[speech] [refer]%%0,2

WANDER==[wandering]%%0,2

WANDER==[modifier] [wandering]%%0,2

WITHX==[with] [subject]%%0,2

WITHX==[subject] [post_with]%%0,2

FORGETX==[forget] [forget_thing]%%0,2

FORGETX==[forget_thing] [forget]%%0,2

FORGET==[forget]%%0,2

FORGETFL==[forgetful]%%0,2

S_CONCERN==[concern] [dementia]%%0,2

S_CONCERN==[dementia] [concern]%%0,2

RISK==[risk] [dementia]%%0,2

RISK==[dementia] [risk]%%0,2

EXAM==[exam]%%0,2

BOOK==[book]%%0,2

HAL_EXCL==[hallucination] [not_hallucination]%%0,2

HAL_EXCL==[not_hallucination] [hallucination]%%0,2

EXM_EXCL==[exam] [normal]%%0,2

EXM_EXCL==[normal] [exam]%%0,2

S_EXCL==[strong_exclude]%%0,2

W_EXCL==[weak_exclude]%%0,2

ICD_INCL==[include_icd]%%0,2

ICD_EXCL==[exclude_icd]%%0,2

HALLUC==[hallucination]%%0,2

S_HALLUC==[modifier] [hallucination]%%0,2

EXCLUDE==[exclude_forget]%%0,2

NEGATE==[negating]%%0,2

AMBIG==[ambig]%%0,2

AMBIG==[ambig_drug]%%0,2

OTH_EXCL==[subject] [strong_exclude]%%0,2

OTH_EXCL==[subject] [weak_exclude]%%0,2

BOI_INCL==[boilerplate]%%0,2

SUBJECT==[subject]%%0,2

CONCENTRATE==[decline] [concentrate]%%0,2

CONCENTRATE==[concentrate] [decline]%%0,2

DEMENTIA==[modifier] [dementia]%%0,2

CALLED==[subject] [call]%%0,2

CALLED==[call] [subject]%%0,2

DECLINE_CALL==[call] [decline]%%0,2

EARLY==[early] [dementia]%%0,2

SENILE==[senile]

HEADACHE==[concern] [brain_injury]%%0,2

HEADACHE==[headache]%%0,2

DENIAL==[denial]%%0,2

PLAN==[subject] [with_plan]%%0,2

BURDEN==[burden] [subject]%%0,2

OTHER_SAYS==[subject] [say]%%0,2
